# Correction: Decreased risk of underdosing with continuous infusion versus intermittent administration of cefotaxime in patients with sickle cell disease and acute chest syndrome

**DOI:** 10.1371/journal.pone.0319527

**Published:** 2025-02-12

**Authors:** Keyvan Razazi, Enora Berti, Jerome Cecchini, Guillaume Carteaux, Anoosha Habibi, Pablo Bartolucci, Romain Arrestier, Ségolène Gendreau, Nicolas de Prost, Anne Hulin, Armand Mekontso Dessap

The eleventh author’s initials are indexed incorrectly in PubMed. The correct initials are: Mekontso Dessap A.
