# Supplementary material for: Decreased risk of underdosing with continuous infusion versus intermittent administration of cefotaxime in patients with sickle cell disease and acute chest syndrome
Source: PLoS One. 2024 Apr 18;19(4):e0302298. doi: 10.1371/journal.pone.0302298 (PMC11025818; doi:10.1371/journal.pone.0302298)
Supplement: Attachment — Submitted filename: HagarReview-PONE-D-21-21735.docx [file pone.0302298.s001.docx]

6/14/2022

The authors have conducted a prospective study comparing intermittent administration of cefoxatime versus continuous infusion for in adults diagnosed with acute chest syndrome (ACS). This is a nearly unique study about the administration of antibiotics in a critically important complication of sickle cell disease. Acute chest syndrome is one of the leading causes of death in hospitalized patients with sickle cell. Sickle cell disease has historically been hard to study, with most studies failing because of lack of enrollment. So any good data is important to get out into the accessible literature. The conclusion that a mainstay of treatment for a life-threatening complication may not be effective because of the delivery mention is important, interesting, and relevant. Even though many centers don’t use cefoxatime as their first-line antibiotic, the same concerns of hyperfiltration and pulse versus continuous dosing requires those centers to consider if their protocols may need adjusting. The authors are commended for a clean study, with an appropriate discussion of limitations.

In short, this study, though limited, is an important addition to prior work that may be exposing a serious flaw in the treatment of a life-threatening condition.

Major comments

1. The acknowledged key limitation is the difference between statistical and clinical significance. Even though the intermittent administration dose had lower troughs, there was no clinical difference. Since not all ACS has bacterial involvement, it likely would take more outcomes to separate a difference. The authors make a compelling case for further studies.
2. The study design implied that only the first thirty patients from each study period were enrolled. Where there patients with ACS not enrolled during these periods. If not, why not. The more data the better. Also were there any repeat patients that got both intermittent and continuous infusions of cefotaxime? Table 1 shows a high percentage of “Previous ACS.” If there were overlap, a brief separate paired analysis would be of interest.
3. The timing of the trough lab values is critical. Was there an enforced standard to ensure the troughs were drawn within an acceptable window.
4. The range of cefotaxime trough level concentrations in the intermittent administration group was 0 - 0 mg/l, then later reported that 17% were in AI was over the 2 mg/L cutoff. This was confusing.
5. Lastly, if the authors had any information on the cost comparison of the two approaches, this would be invaluable. If the cost, time, and labor of the intermittent administration exceeded the continuous administration, then a compelling case for continuous administration could be made.

Minor comments:

1. Generic drug names are not capitalized.
2. Cefotaxime is not the first-line antibiotics in many centers and when used it is often at a higher dose (60 to 100 mg/kg/day). The limitations (line 190) could possibly add something like “assessment of a single antibiotic at a single dose”
3. Given the n of 300, I’m not sure why a non-parametric test was used. Usually if the n is greater than twenty, more standard parametric tests (t-tests, ANOVA) can be used and are easier to interpret. Although not perfect, several simple tests for normality ( Wilkes, e.g.) can by use to test if the distribution is “normal enough. The authors may want to analyze their data with parametric statistics to see if they get the same conclusion. This would make their conclusion more robust.
4. I wasn’t sure why the categorical variables were changed to a percentage before Chi-2 testing. Usually the raw counts are tested. Any mathematical manipulation changes the information in the data. Again, they may want to perform the Chi-2 testing on the raw data for robustness.
