# Supplementary material for: Decreased risk of underdosing with continuous infusion versus intermittent administration of cefotaxime in patients with sickle cell disease and acute chest syndrome
Source: PLoS One. 2024 Apr 18;19(4):e0302298. doi: 10.1371/journal.pone.0302298 (PMC11025818; doi:10.1371/journal.pone.0302298)
Supplement: Attachment — Submitted filename: reponse reviewer 06 09 AMD.docx [file pone.0302298.s002.docx]

The authors have conducted a prospective study comparing intermittent administration of cefoxatime versus continuous infusion for in adults diagnosed with acute chest syndrome (ACS). This is a nearly unique study about the administration of antibiotics in a critically important complication of sickle cell disease. Acute chest syndrome is one of the leading causes of death in hospitalized patients with sickle cell. Sickle cell disease has historically been hard to study, with most studies failing because of lack of enrollment. So any good data is important to get out into the accessible literature. The conclusion that a mainstay of treatment for a life-threatening complication may not be effective because of the delivery mention is important, interesting, and relevant. Even though many centers don’t use cefotaxime as their first-line antibiotic, the same concerns of hyperfiltration and pulse versus continuous dosing requires those centers to consider if their protocols may need adjusting. The authors are commended for a clean study, with an appropriate discussion of limitations.

In short, this study, though limited, is an important addition to prior work that may be exposing a serious flaw in the treatment of a life-threatening condition.

We sincerely thank the reviewer for their constructive comments. All comments have been addressed and changes made in the manuscript. We have submitted a point by point response to all reviewers comments. We feel those changes improved the manuscript.

Sincerely

Major comments

1. The acknowledged key limitation is the difference between statistical and clinical significance. Even though the intermittent administration dose had lower troughs, there was no clinical difference. Since not all ACS has bacterial involvement, it likely would take more outcomes to separate a difference. The authors make a compelling case for further studies.

Response: We agree with reviewer’s comment. In accordance, we added a sentence in the limitation section of the discussion as follows: Further studies are also warranted to assess the effect of the optimization of pharmacokinetic/pharmacodynamic parameters on clinical outcomes in this setting, inasmuch as the rate of bacterial documentation is low during ACS.

2. The study design implied that only the first thirty patients from each study period were enrolled. Where there patients with ACS not enrolled during these periods. If not, why not. The more data the better. Also were there any repeat patients that got both intermittent and continuous infusions of cefotaxime? Table 1 shows a high percentage of “Previous ACS.” If there were overlap, a brief separate paired analysis would be of interest.

Responses: There is no overlap between periods. Only two patients got the two infusion methods, during two separate ACS episodes, precluding the use of a paired analysis in this subgroup. Dosage results for those two patients were zero in intermittent infusion period and over the 2 mg/L cutoff (21.7 and 6.3 mg/l) in the continuous infusion period. In accordance with reviewer’s comment, a flow chart was provided in the manuscript supplement and the result section was modified as follows: “Sixty consecutive episodes of severe acute chest syndrome (30 in each group) with a dosage of cefotaxime were assessed in 58 adult patients with sickle cell disease (see flow chart in online supplement)”.

3. The timing of the trough lab values is critical. Was there an enforced standard to ensure the troughs were drawn within an acceptable window.

Response: As mentioned in the methods section, for the intermittent administration period, blood samples were obtained just before infusion of the drug (trough concentrations). We agree with reviewer that a dosage sampled not just before the infusion could falsely increase the concentration, but in this period most concentrations (n=25; 85%), were <2mg/L . As mentioned in the methods section, for the continuous infusion period, blood samples were obtained after 24 hours of infusion.

4. The range of cefotaxime trough level concentrations in the intermittent administration group was 0 - 0 mg/l, then later reported that 17% were in AI was over the 2 mg/L cutoff. This was confusing.

Response: 0 - 0 mg/l refers to the interquartile range. More than 75% of patients (n=25, 85%) had a concentration of 0, explaining why the 1^st^ and 3^rd^ quartile were 0.

5. Lastly, if the authors had any information on the cost comparison of the two approaches, this would be invaluable. If the cost, time, and labor of the intermittent administration exceeded the continuous administration, then a compelling case for continuous administration could be made.

Response: The time and associated costs (drug and supply) of administering a continuous versus an intermittent infusion were compared. We observed 10 nurses for each infusion method. The time needed per day to prepare and administer the antibiotic was calculated for each infusion method. Daily nurse timework was lower with continuous infusion as compared to bolus infusion : 234 [234-234] vs 278 [278-556] seconds, p< 0.001. The costs for all items used in drug preparation and administration including syringe pump amortization expenses were also calculated. Cost of all necessary material and drug were lower with continuous infusion as compared to bolus infusion: 6.1 [6.0-6.1] vs 8.2 [7.9-15.9] euros, p< 0.001. Labor cost of the nursing time was not considered.

These results are in accordance with previous study on cost analysis of continuous infusion of other antibiotics [1]. However, we should also consider the limited availability of syringe pump in some low-income countries. These points were added in the manuscript (methods, results, and discussion).

Minor comments:

1. Generic drug names are not capitalized.

Responses: Cefotaxime was changed for cefotaxime

2. Cefotaxime is not the first-line antibiotics in many centers and when used it is often at a higher dose (60 to 100 mg/kg/day). The limitations (line 190) could possibly add something like “assessment of a single antibiotic at a single dose”

Response: in accordance with reviewer comment, we added the following sentence as a limitation: “Our study has several limitations, including the assessment of a single antibiotic at a given dose”. We also added the following data in the result section: “There was no significant correlation between trough concentration and antibiotic daily dose (Spearman’s Rho = 0.16, p=0.84).”

3. Given the n of 30, I’m not sure why a non-parametric test was used. Usually if the n is greater than twenty, more standard parametric tests (t-tests, ANOVA) can be used and are easier to interpret. Although not perfect, several simple tests for normality ( Wilkes, e.g.) can by use to test if the distribution is “normal enough. The authors may want to analyze their data with parametric statistics to see if they get the same conclusion. This would make their conclusion more robust.

Response: As many concentration were 0 in the intermittent infusion group, the distribution was not normal, with a p value for Kolmogorov-Smirnov test < 0.001

4. I wasn’t sure why the categorical variables were changed to a percentage before Chi-2 testing. Usually the raw counts are tested. Any mathematical manipulation changes the information in the data. Again, they may want to perform the Chi-2 testing on the raw data for robustness.

Response: Sorry if the sentence was not understandable. Categorical variables were not transformed; we corrected this point in the statistical section.

1. Florea NR, Kotapati S, Kuti JL, Geissler EC, Nightingale CH, Nicolau DP. Cost analysis of continuous versus intermittent infusion of piperacillin-tazobactam: a time-motion study. Am J Health-Syst Pharm AJHP Off J Am Soc Health-Syst Pharm. 2003;60:2321–7.
