# Supplementary material for: Decreased risk of underdosing with continuous infusion versus intermittent administration of cefotaxime in patients with sickle cell disease and acute chest syndrome
Source: PLoS One. 2024 Apr 18;19(4):e0302298. doi: 10.1371/journal.pone.0302298 (PMC11025818; doi:10.1371/journal.pone.0302298)
Supplement: Attachment — Submitted filename: Responses to Reviewer R4.docx [file pone.0302298.s003.docx]

We thank the reviewer for their constructive comments. All comments have been addressed and changes made in the manuscript. We have submitted a point-by-point response to all reviewers’ comments.

All authors have read and approved the submission of the manuscript, and the manuscript has not been published and is not being considered for publication elsewhere in whole or in part in any language. Please, feel free to contact me for any requirements related to this submission.

Thank you so much for your attention.

Sincerely,

Keyvan Razazi, MD

Corresponding Author

Service de Réanimation Médicale, Hôpital Henri Mondor, Créteil, France

E-mail : keyvan.razazi@aphp.fr

Sincerely

Reviewer #5: Here are some feedback and improvement suggestions for the article titled "Decreased risk of underdosing with continuous infusion versus intermittent administration of cefotaxime in patients with sickle cell disease and acute chest syndrome" with line numbers:

**Abstract:**
The study population could be described more specifically with age range and number of participants.

Responses : In accordance with reviewer’s comment, a sentence was added as follows: We included 60 episodes of acute chest syndrome in 58 patients (29 [25-34] years, 37/58 (64%) males).

**Introduction:**
The limitations of intermittent cefotaxime dosing and the potential consequences of underdosing could be better contextualized. The rationale for the study could be more clearly stated.

Responses: In accordance with reviewer’s comment, the rationale was further detailed as follows: ß-lactams are the most commonly prescribed antimicrobial agents in the critically-ill, especially in patients with acute lung injury [4] . ß-lactam antibiotics display time-dependent activity, where bacterial killing (microbiologic cure) and treatment efficacy (clinical cure) correlate with the duration of time that free (unbound) plasma drug concentrations remain above the minimal inhibitory concentration of the offending pathogen (*fT*>MIC). These characteristics suggest optimal effects with continuous infusion (CI) rather than traditional intermittent administration (IA) dosing. IA dosing may result in ß-lactam concentrations below the MIC for much of the dosing interval, increasing the risk of treatment failure and emergence of resistant strains. In patients with sepsis, interventions aimed at optimising antibiotic therapy demonstrated the greatest improvements in clinical outcomes [4,5].

**Methods:**
The recruitment process for patients could be described in more detail.
It should be clarified whether patients were randomized or not.
More information on the dosage regimen for each group could be provided.
It should be clarified whether the study was double-blinded or not.

Responses: This before-after study was not randomized and was not double blinded. In accordance with reviewer’s comment, these points were further detailed in the methods as follows: In this before-after study, consecutive patients were included without randomization.

The dosage regimen was also further detailed, as follows:

The target dose of cefotaxime was 60 mg/kg/day for 7 days (or less according to procalcitonin concentrations) in both groups. IA used 30-60 min intravenous perfusions q6-8 hours [1g q8 hours (n=10); 2g q8 hours (n=7); 1g q6 hours (n=5); 1g q4 hours (n=8)]; , while CI used a 12-h infusion with an electric syringe pump, after a loading dose of 1g over 60 minutes.

The statistical methods used for data analysis could be described in more detail.

Responses: In accordance with reviewer’s comment, a sentence was added as follows:

Correlations were tested using the Spearman’s method.

**Results:**
The difference in incidence of underdosing between the two groups should be clarified.

The numerical values for the percentages of underdosing in each group should be provided.

Responses: In accordance with reviewer’s comment, numerical values and percentages were added in results section as follows:

Episodes with undetectable (<2 mg/L) trough concentrations were more frequent in IA group (n=25, 83%) than in CI group (n=2; 7%), p<0.01.

More detail on the adverse events observed in the study could be given.

Responses: In accordance with reviewer’s comment, adverse events were detailed as follows: We observed no adverse event associated with cefotaxime use, either neurologic (seizure, encephalopathy, coma, or delirium), hematological, or renal.

Discussion:
The limitations of the study, including the small sample size and potential biases, should be discussed in more detail.

Responses: In accordance with reviewer’s comment, limitations on sample size were added:

Our study has several limitations, including the small sample size, the assessment of a single antibiotic at a given dose, and the lack of clinical outcome. In addition, the before-after design and monocenter setting in an intensive care unit may infer several bias. Our results cannot be extrapolated to SCD patients with decreased renal function, to other antibiotics with a concentration-dependent therapeutic effect such as aminoglycosides or to patients without SCD.

The clinical implications of the study results and potential for improving patient outcomes should be discussed in more detail.

Responses: In accordance with reviewer’s comment, we added this point as follows: Our findings have major clinical implications, given the burden of severe infections and difficult to treat infections in patients with SCD [21,22].

Conclusion:
The conclusion could be more clearly stated with a summary of the study results and their clinical significance.

Responses: In accordance with reviewer’s comment, the conclusion was changed as follows:

As compared to IA, CI of cefotaxime maximizes the pharmacokinetic/pharmacodynamic parameters. The median residual concentration was higher in the CI than IA group. CI of time-dependent antibiotics seems to decrease the risk of underdosing in patients with SCD. Daily nurse timework and cost were also lower with CI. Further studies are needed to assess the impact of CI on clinical outcomes in patients with SCD.

References: Some references are missing information such as authors' names or full journal titles, and some are not in proper citation format. These should be corrected.

Responses: We used the plos one style in Zotero

Improvement suggestions: The introduction could be strengthened with more background information on ACS in SCD, its prevalence, and clinical management.

Response: In accordance with reviewer suggestion, introduction was strengthened with data on prevalence of SCD and ACS, as well as clinical management, as follows:

Sickle-cell disease (SCD) is one of the most common severe monogenic disorders worldwide. Approximately, 300,000 infants are born annually worldwide with SCD and 100,000 people have SCD in the US [1].

Acute chest syndrome (ACS) is a major complication of SCD and the first cause of mortality in adult patients [2]. ACS is characterized by fever and/or respiratory symptoms with new pulmonary infiltrates. Bacterial infection is documented in only a minority of cases [2,3]. However, empirical antibiotic therapy is often used in ACS because establishing a definitive etiology is not always possible.

The discussion could benefit from a comparison of the current study's results with other studies in the literature on cefotaxime dosing in SCD patients with ACS.

Response: In accordance with reviewer’s comment, our study was compared to the only study from the literature on cefotaxime dosing in patients with SCD as follows:

Our results are in accordance with those of Maksoud et al who found very low attainment of target cefotaxime serum concentrations in pediatric SCD patients (only 5.6% of patients achieved 90% of time above the MIC of 2mg/L) [23].

A limitation of the study is that it only included patients with SCD and ACS, so the generalizability of the results to other patient populations may be limited. This limitation could be acknowledged and discussed in the discussion section.

Response: In accordance with reviewer’s comment, we added theses points as follows: Most patients presented with augmented renal clearance, a frequent feature in patients with SCD at baseline and during ACS. Our results cannot be extrapolated to SCD patients with decreased renal function, to other antibiotics with a concentration-dependent therapeutic effect such as aminoglycosides or to patients without SCD.

The article could benefit from a clearer explanation of the clinical implications of the study results for patient care and potential areas for future research.

Response: In accordance with reviewer’s comment, we added this point as follows:

Our findings have major clinical implications, given the burden of severe infections and difficult to treat infections in patients with SCD [21,22].

Consider reorganizing the methods section to improve clarity and readability.

Response: In accordance with reviewer’s comment, we reorganized the methods section with sub-sections for more clarity as follows:

Consider adding a section on future directions and potential avenues for further research.

Response: In accordance with reviewer’s comment, we added these points as follows in the discussion section: Further studies are needed to assess the feasibility of this approach in medical wards. Further studies are also warranted to assess the effect of the optimization of pharmacokinetic/pharmacodynamic parameters on clinical outcomes in this setting, inasmuch as the rate of bacterial documentation is low during ACS. Our findings suggests exploring the pharmacokinetic/pharmacodynamic of other antibiotics (especially for bloodstream and difficult to treat infections like bone-joint infections [20]) and that of other treatments influenced by renal clearance (e.g., morphine) in patients with SCD.
