# Supplementary material for: Decreased risk of underdosing with continuous infusion versus intermittent administration of cefotaxime in patients with sickle cell disease and acute chest syndrome
Source: PLoS One. 2024 Apr 18;19(4):e0302298. doi: 10.1371/journal.pone.0302298 (PMC11025818; doi:10.1371/journal.pone.0302298)
Supplement: Attachment — Submitted filename: r7 responses to reviewers AMD.docx [file pone.0302298.s004.docx]

PONE-D-21-21735R7

Decreased risk of underdosing with continuous infusion versus intermittent administration of Cefotaxime in patients with sickle cell disease and acute chest syndrome.

PLOS ONE

We thank the reviewer for their constructive comments. All comments have been addressed and changes made in the manuscript. We have submitted a point-by-point response to all reviewers’ comments.

We would like to bring to the editor's attention that it has now been two and a half years since this paper has been submitted to your journal, with a total of eight reviewing sessions involving at least ten reviewers, and only minor corrections requested in the last round. Therefore, we deeply hope that at this very advanced stage of the reviewing process, after 30 months, an ultimate and favorable decision will be made.

All authors have read and approved the submission of the manuscript, and the manuscript has not been published and is not being considered for publication elsewhere in whole or in part in any language. Please, feel free to contact me for any requirements related to this submission.

Thank you so much for your attention.

Sincerely,

Keyvan Razazi, MD

Corresponding Author

Service de Réanimation Médicale, Hôpital Henri Mondor, Créteil, France

E-mail : keyvan.razazi@aphp.fr

Sincerely

Overall: The authors chose to conduct a study which evaluated the effect of continuous infusion over intermittent administration of cefotaxime in patients with sickle cell disease who have ACS. The authors specifically evaluated the trough level of cefotaxime, which was significantly better after CI. Although the authors did not see a clinical benefit, they reported a time-saving benefit, and a cost-benefit, whose relevance to practical use is questionable owing to the benefit being extremely small.

I understand that this paper has been reviewed by several reviewers before me and has been revised seven times. Therefore, and to the authors' credit, the quality of the manuscript was acceptable, and I only have small comments to offer. Although the authors chose to evaluate a very narrow, and specific, aspect of drug dosing, nevertheless, they have produced research which is not erroneous. And although there are no clinical benefits of this study, the authors have indeed answered a clinical question which hitherto had not been addressed before.

 
***Abstract***

[1] Page 3. Under the "Patients" subheading, the authors currently report the number of severe ACS "episodes" they encountered. Although this is relevant given the study was centered around these episodes, the author should also quote the number of patients as they do under the next subheading.

Responses: In accordance with reviewer’s comment, the number of patients was added in patients suheading as follows: “Sixty consecutive episodes of severe acute chest syndrome in 58 adult patients with sickle cell disease.”

[2] Page 4. The authors conclude that CI helped cefotaxime achieve a better pharmacokinetic/pharmacodynamic profile. While this is true, and reflects the original purpose of the study, it is advisable that the authors also highlight that this did not translate into better clinical outcomes when compared to IA (similar hospital stays etc.). This would help future readers understand the wider picture without having to dig for data within the manuscript.

Responses: In accordance with reviewer’s comment, a sentence was added in the conclusion of the abstract as follows: “The clinical outcome did not differ between the two administration methods; however, the study was underpowered to detect such a difference.”

***Introduction***

[1] I have no particular comments for this section. The introduction flows well and is well-written. Although this is not a strict comment, but I would have liked to see the authors highlight other drugs which are also employed in ACS. A sentence about their use, and then the rationale for choosing cefotaxime would have been interesting to see. As of now, the introduction does justice to the research question at hand.

Responses: In accordance with reviewer’s comment , a sentence was added as follows: “In clinical practice, empirical intravenous antimicrobial therapy is usually a combination therapy with a macrolide targeting intracellular bacteria and a beta lactam targeting pyogenic bacteria (amoxicillin in mild ACS and cephalosporin for severe ACS) [4].”


***Methods***

[1] Page 9. Time and Cost. Currently, the authors simply state that they calculated the cost of instruments, and labor etc.; and the time needed by observing ten nurses. However, this does not convey how this was achieved. As a researcher who would like to replicate this study, it offers no information on how this data was collected. As of now, we just know that it was collected. The authors should consider adding sentences that state all this data was collected. For example, what instruments were considered when calculating the cost (infusion pump? medicines? et al.), did the authors review institutional records to identify the cost? If so, it should be stated. When calculating the time spent, who recorded the time that was taken? And how?

I hope the authors understand that one of the pillars of rigorous research is reproducibility, which requires that all information regarding the research should be available. Therefore, to make this paper more scientifically accurate, while it may sound inconsequential, and to maintain scientific rigor it is necessary that such details be highlighted.

Responses: As requested by the reviewer, the methods for time and costs were detailed in the methods section and in the online supplement as follows:

“The time and associated costs (drug and consumables) of administering a continuous versus an intermittent infusion were compared. One author (KR) observed 10 nurses administering, in succession, in a simulation room, a continuous and an intermittent infusion in a cross-over design (five nurses began by the continuous infusion while five others began by the intermittent infusion). The time needed to prepare and administer the antibiotic was calculated for each infusion method (see online supplement). This time was collected using a stopwatch, and included the preparation phases in the patient's room, tubing connection, start of infusion, stop of infusion, and tubing disconnection. The time dedicated to collecting equipment was not taken into account because most of the material needed was located in the room. The time needed per day to prepare and administer the antibiotic was computed as the product of the time for one administration by the number of administrations per day.

The costs for all items used in drug preparation and administration including syringe pump amortization expenses were also calculated (see online supplement). The costs were identified by reviewing institutional records (tax included). These costs included: cefotaxime, crystalloid sodium 0,9% 50ml, transfert needle, sterile dressings, syringe with luer lock, extension set (only for continuous infusion) or infusion set (only for intermittent administration), and syringe pump amortization expenses (only for continuous infusion). Labor cost of the nursing time was not considered.”

Of note, when revising the costs, we corrected a calculating error, not affecting the trend of the results: Cost of all necessary material and drug were lower with CI as compared to IA: 6.1 [6.1-7.0] vs 7.8 [5.6-11.1] euros, p<0.01. Sorry for this mistake.

***Discussion***

[1] Page 15. The authors say "Moreover, nurse timework and costs were decreased with CI as previously published with other antibiotics". The authors should consider discussing whether saving 44 seconds by using CI provided an actual clinical/logistical benefit or not, as well as its importance.

Response: In accordance with reviewer’s comment, we added the following sentences: “The time saved for nurses was due to the reduction in the number of administrations per day. However, this result was obtained in a simulation room, and the difference was numerically marginal (less than one minute).”

***Conclusion***

[1] Page 16. Conclusion. The authors do not state that there was no clinical benefit of CI over IA.

Response: In accordance with reviewer comment, a sentence was added in the conclusion of the manuscript as follows: “The clinical outcome did not differ between the two administration methods; however, the study was underpowered to detect such a difference.”
